# Early functional changes in lewy body dementia: roles of dynamics, locus coeruleus, and compensation

**DOI:** 10.1186/s13195-025-01828-1

**Published:** 2025-08-23

**Authors:** Kristína Mitterová, Eva Výtvarová, Anežka Kovářová, Martin Lamoš, Jan Fousek, Irena Rektorová

**Affiliations:** 1 Central European Institute of Technology (CEITEC), Masaryk University, Pekařská 53, Brno, 65691 Czech Republic; 2 Faculty of Medicine, Masaryk University, Pekařská 53, Brno, 65691 Czech Republic; 3 1st Department of Neurology, St. Anne’s University Hospital, and Faculty of Medicine, Masaryk University, Brno, Czech Republic

**Keywords:** Dementia with lewy bodies, Prodromal dementia with lewy bodies, Dynamic functional connectivity, Locus coeruleus, Cognitive reserve, Fluidity, Node strength

## Abstract

Dementia with Lewy bodies (DLB) is marked by multidomain cognitive impairments, with fluctuations in cognition and alertness being among the most common clinical features. Disruptions in functional connectivity are thought to underlie these fluctuations, but it remains unclear whether such patterns are already present at prodromal stages. We investigated the presence of static and dynamic functional connectivity alterations and their contribution to the clinical phenotype of prodromal DLB, and explored their association with declining locus coeruleus integrity, while considering premorbid intelligence (a proxy for cognitive reserve) as a moderating factor. Three groups of participants on the prodromal spectrum were analyzed: 29 healthy controls, 58 cognitively normal subjects with core clinical features of prodromal DLB (CN-CCF), and 39 subjects with mild cognitive impairment due to Lewy body dementia (MCI-LB). Dynamic and static functional connectivity features were derived from resting-state source-reconstructed high-density EEG, and integrity of the right caudal locus coeruleus was quantified using neuromelanin-sensitive MRI. Robust analyses, such as PERMANOVA, Spearman correlations and general linear models were conducted to study the relationships. We observed a nonlinear trajectory of two functional connectivity metrics—temporal variability (fluidity) and connectedness (average node strength)—across symptom severity, especially in the delta frequency band. Both measures (*F*(2,123) = 1.86, *p* =.037; *F*(2,123) = 1.51, *p* =.023, respectively) were elevated in the intermediate severity groups—that is, in CN-CCF and possible MCI-LB (i.e., one core clinical feature)—and this was associated with better executive functioning after controlling for age and premorbid intelligence (rho = 0.26, *p* =.004; rho = 0.28, *p* =.002, respectively). Additionally, elevated fluidity (rho = − 0.34, *p* =.034) and average node strength (rho = − 0.37, *p* =.022) were also correlated with fewer fluctuations in alertness in subjects with MCI-LB. Fluidity was further associated with right caudal locus coeruleus integrity, particularly among individuals with lower premorbid intelligence (*F*(5,97) = 3.56, *p* =.005). Our findings indicate that increased dynamic reconfiguration and enhanced connectivity may serve compensatory functions in early DLB, helping temporarily preserve cognition. As DLB progresses, these mechanisms wane, with noradrenergic contributions more evident in individuals with lower cognitive reserve.

**Clinical trial number** Not applicable.

**Supplementary Information:**

The online version contains supplementary material available at 10.1186/s13195-025-01828-1.

## Introduction

Dementia with Lewy bodies (DLB) is characterized by multidomain cognitive impairments, particularly affecting visuoperceptual and executive functions from early onset [1]. Executive functions provide means for temporary storage, information binding, and integration; deficits impact a wide range of functions, including learning and memory [2]. Mild cognitive impairment with Lewy bodies (MCI-LB), a pre-dementia stage marked by preserved self-sufficiency, is associated with gradual cognitive decline accompanied by noticeable noncognitive symptoms (i.e., core clinical features [1]). Among these, fluctuating cognition and alertness affect up to 90% of patients [1, 3, 4].

It has been proposed that fluctuations are accompanied by disrupted brain connectivity [5], which may arise from disordered switching between brain states [6]. Although the precise localization of connectivity disruptions is still debated [8–9], graph-theoretical network metrics suggest that the functional connectivity (FC) of distant brain regions in DLB is desynchronized [10]. In addition, growing evidence suggests that α-synuclein accumulation contributes to large-scale network disruption [11, 12]. It is not yet known whether disrupted pattern of FC is already present in MCI-LB. To address this, we employed EEG, which has consistently revealed a typical slowing of dominant EEG rhythms in DLB [13] and MCI-LB [14].

Static connectivity measures neglect the temporal variability of FC, which may be important for understanding the early changes in the temporal dynamics of processes, e.g., frequency slowing of oscillations [15]. Prior research suggests that one factor contributing to cognitive decline and fluctuations may be an increased temporal rigidity of the brain networks, reflected in reduced variability in FC efficiency [16]. In other words, the functional network dynamics in DLB is less variable than in healthy aging, where functionally related regions cooperate in spontaneous low-frequency fluctuations supporting metabolically effective operations [17]. Optimal cognitive functioning requires dynamic shifting between states, and individuals with more dynamic networks were shown to perform better in behavioral tasks involving episodic memory, sustained attention, self-regulation [18], and working memory [19].

In parallel, growing evidence indicates that the locus coeruleus (LC), a brainstem nucleus, is essential for regulating arousal and attentional flexibility, thereby playing a role in cognitive functioning. The loss of neuromelanin-rich neurons in the LC disrupts norepinephrine production in the central nervous system, contributing to cognitive symptoms [20], particularly lapses of attention and attention deficits [21, 22] and visual memory deficits [23]. LC integrity, assessed non-invasively using neuromelanin-sensitive MRI sequences, was shown to deteriorate more in subjects with MCI-LB than in healthy elderly subjects [23], and its degeneration even precedes that of the substantia nigra in Parkinson’s disease [24]. The reduced structural integrity of LC has been linked to cognitive decline in healthy aging; conversely, preserved LC is suggested to play a role in delaying the symptoms of cognitive decline [25, 26]. Here we study, for the first time, resting-state temporal variability (dynamic functional connectivity, dFC) alongside static FC (sFC) across different frequency bands in three groups along the prodromal DLB (pDLB) spectrum: healthy controls (HC) with subjective complaints consistent with prodromal DLB, cognitively normal individuals manifesting core clinical features of DLB (CN-CCF), and MCI-LB. This design allows us to study a continuous trajectory, bridging the empirical research gap between subjective impairment [27] and developed DLB [28], and to probe whether CN-CCF represents a transitional or compensatory phase. While MCI-LB is a well-characterized prodromal stage with cognitive decline, CN-CCF group offers insight into an understudied population with early pathophysiological changes manifesting with symptoms like REM sleep behavior disorder or cognitive fluctuations. We treat CN-CCF as an intermediate stage between subjective clinical impairment and MCI-LB to assess whether early changes in functional connectivity and LC integrity are already detectable, reflecting compensation, disruption, or both.

It has been well described by contemporary aging theories that, beyond age-related brain atrophy and functional alterations, the brain undergoes compensatory changes by recruiting additional regions (e.g., *The Scaffolding Theory of Aging and Cognition*) [29] or reorganizing functional networks [30]. Growing evidence suggests that these principles may also apply to prodromal degenerative diseases, where some individuals present with better-than-expected cognitive performance alongside altered FC topology. Previous neuroimaging studies have reported several brain features associated with greater cognitive resilience, such as increased topological segregation of functional brain networks in Alzheimer’s disease [31], and parieto-premotor cortical compensation in Parkinson’s disease, associated with lower severity of cognitive symptoms and bradykinesia [32].

Our goal is to investigate the association of early sFC and dFC changes with pDLB-related pathology and cognitive decline, controlling for potential mechanisms of premorbid intelligence and education, known determinants of the level of cognitive functioning in older age and established proxies of the cognitive reserve concept [33, 34]. This will aid in evaluating the unique contribution of sFC/dFC network alterations on cognitive decline. Concerning sFC, we focused on quantifying global connectedness by the average node strength, mesoscale structure by the modularity coefficient, and functional integration and segregation by normalized average characteristic path length and normalized average clustering coefficient. In line with previous research [35], we hypothesized that global connectedness is higher in healthy controls (HC) than in MCI-LB, and that it is positively associated with cognitive functions across the continuum of HC to MCI-LB. To assess dFC, we used fluidity, a measure of spontaneous activity that captures the recurrence of co-activation patterns over time [27, 36, 37]. Following previous studies [16, 27], we hypothesized that fluidity is lower in MCI-LB than in HC, following a trajectory of cognitive decline, and that it is positively related to cognitive performance and inversely related to fluctuations of alertness. Lastly, we hypothesized that lower LC integrity (i.e., lower neuromelanin signal intensity) is associated with temporal rigidity of the dFC (operationalized as fluidity), reflecting a cascade of disrupted noradrenaline neurotransmission previously linked to the fluctuating cognition characteristic for DLB [5].

## Methods

### Participants

Participants were recruited from the community through advertisements and screened via telephone to assess the inclusion and exclusion criteria. Inclusion criteria were age between 55 and 70 years, and subjective symptoms of MCI-LB. Exclusion criteria included severe medical conditions, major brain injury, other psychiatric or neurological disorders, and MRI contraindications. After the phone interview, participants underwent clinical, neuropsychological, and neurocognitive evaluations conducted by neuropsychologists and a neurologist. Based on these assessments, participants were classified according to the presence of core clinical symptoms of prodromal DLB [1]. Subsequently, they underwent high-density resting-state EEG and neuromelanin-sensitive MRI (described in detail below), completed across two visits.

This study was approved by the Masaryk University Research Ethics Committee (approval number EKV-2022-094) and conducted in accordance with the Declaration of Helsinki and relevant ethical guidelines. All participants provided written informed consent before data acquisition. Clinical trial number: not applicable.

### Neuropsychological and clinical testing

All subjects were classified into three groups based on the following combination of cognitive and noncognitive symptoms of prodromal DLB described by McKeith et al. [1].

- Cognitive symptoms: Performance on two or more cognitive tests below − 1 SD of the age-appropriate norm (MCI+); subjects without objective cognitive impairment were considered cognitively healthy (MCI-).
- Noncognitive symptoms: At least one of the core clinical features (CCF+) of DLB. The presence of CCFs was evaluated using these scales: MDS-UPDRS III – Unified Parkinson’s Disease Rating Scale (cut-off ≥ 4 points); NPI – Neuropsychiatric Inventory Questionnaire - hallucination subscale (cut-off = 1 point); MFS – Mayo Fluctuations Scale (cut-off ≥ 3 points); RBDq – REM Sleep Behavior Disorder Questionnaire (cut-off ≥ 5 points).

The three resulting classification groups were: HC (MCI-, CCF-; *n* = 39), CN-CCF (MCI-, CCF+; *n* = 58), and MCI-LB (MCI+, CCF+; *n* = 39). For the purpose of exploring relationships with selected outcome variables, we split the MCI-LB group based on severity—the number of core clinical features [1, 38]—into possible MCI-LB (one core clinical feature; *n* = 22) and probable MCI-LB (two or more core clinical features; *n* = 16). Of the 39 MCI-LB subjects, one was unclassified as possible or probable due to missing data. Two patients had Levodopa Equivalent Doses of 352 mg and 320 mg. No participants in this study were taking antipsychotic medication at the time of assessment. Subjects with undefined MCI (MCI+, CCF-) were excluded from the study (*n* = 15).

Four other clinical scales were used to supplement the neuropsychological assessment of the subjects: MoCA – Montreal Cognitive Assessment; UPSIT – University of Pennsylvania Smell Identification Test; ESS – Epworth Sleepiness Scale; and GDS – Geriatric Depression Scale.

### Blood biomarkers

Plasma samples were collected from all participants via venipuncture to complement the subjects’ clinical characteristics, particularly to provide additional context on the presence of co-pathologies. Levels of plasma glial fibrillary acidic protein (GFAp), neurofilament light chain (NfL), and pTau epitopes were analyzed by the Clinical Neurochemistry Laboratory at the University of Gothenburg, Sweden, using the Simoa HD-1 platform (Quanterix, Billerica, MA, USA) in a blinded setup, following previously established protocols [39]. Plasma biomarker positivity counts were based on the reported cut-off scores from ROC analyses (≥ for positivity): NfL: 17.58 pg/ml [40], pTau181: 12.2 pg/ml [41], pTau217: 2.50 pg/ml [42], pTau231: 17.652 pg/ml [43].

### Neuromelanin-sensitive MRI acquisition and processing of LC

We used the same processing pipeline for neuromelanin-sensitive MRI (NMS-MRI) as in previous studies [23, 26]. First, we skull-stripped T1-weighted images using ANTs software, followed by spatial normalization to the MNI space, and coregistered the NMS-MRI image to the T1-weighted image in native space (using rigid transformations). Finally, we combined all transformations to normalize the LC and pontine tegmentum (PT) masks in one step.

To acquire LC signals, a manually drawn overinclusive mask was used to define the LC search area, and the reference region was set in the central PT, chosen for its stable signal-to-noise ratio. This was done to normalize the intensity variations between subjects and slices. The intensity was adjusted so that the reference region was unified across all images. We then calculated the contrast ratio (CR) between the LC and PT using this formula:

*LC-CR = (LC intensity - PT intensity) / PT intensity.*

We identified the voxel with the highest intensity in both left and right LC and placed a cross mask of five voxels over these peak voxels to capture the signal. If this cross was too close to the fourth ventricle, we shifted the center voxel a step back to avoid signal loss and stay within the region of interest. The signal was measured in three slices representing different parts of the LC. The middle slice was positioned 7 mm below the lower edge of the inferior colliculus, based on past research.

For the purposes of this paper, we selected the right caudal LC based on a previous work indicating that this area was significantly reduced in pDLB compared to HC, and this reduction was significantly related to cognitive performance [23].

### EEG processing

High-density eyes-closed resting-state scalp EEG was recorded in a shielded cabin for a duration of 15 min using an HDEEG Electrical Geodesics, Inc. (EGI GES 400 MR) system with 256 channels, 1 kHz sampling rate, and Cz electrode as a reference. The data processing was conducted in MATLAB and had three steps: preprocessing [44–47], source reconstruction, and connectivity computation. Preprocessing (utilizing the EEGLAB toolbox’s functions [44]) consisted of discarding facial and neck electrodes due to the frequent presence of muscle artifacts [45], filtering to 0.1–100 Hz using a second-order Butterworth filter, 12 dB/octave roll-off, and forward and backward passes to eliminate phase shifts [46], and suppression of ICA-identified eye movement and ECG artifacts (no more than five components were suppressed) [47]. The signals from the same 204 electrodes for each subject were kept, re-referenced to the average reference, and entered into the source reconstruction procedure.

Using Cartool [48] for electrical source imaging, the forward Locally Spherical Model with Anatomical Constraints (LSMAC) model based on the MNI template, and the Low-resolution electromagnetic tomography analysis (LORETA) algorithm [49], the centroids of solution points belonging to the regions of interest defined by the AAL atlas [50] were projected to a refined average orientation [51], leading to representations of activity in the regions of interest. The first seven minutes of the time series were filtered into four frequency bands: delta (δ-band) 0.1–4 Hz, theta (θ-band) 4–6 Hz, alpha (α-band) 6–12 Hz, and beta (β-band) 12–30 Hz. The atypical ranges of the θ-band and α-band were chosen due to the known shift of the alpha power peak to lower frequencies below 8 Hz in DLB [38], with the aim of not splitting this phenomenon into two frequency bands.

The dynamic functional connectivity representation within each frequency band and a broadband of 0.5–60 Hz was based on the circular correlation coefficient (CCor) computed in sliding windows (window length 1s, step 0.1s), as used in Breyton [37]. The CCor is a similarity measure suitable for time series with repeating patterns and is based on the Hilbert transform and phase computations. Taking the instantaneous phase $\:{\phi\:}_{i}\:$and a mean phase $\:\overline{{\phi\:}_{i}}$ within each time window, the circular correlation, that is functional connectivity within a window *t*, is defined as

$$CCo{r}_{i,j}\left(t\right)\:=\:\frac{{\sum\limits_{t\epsilon\tau_{win}}}\,\text{sin}\left({\phi\:}_{i}\left(t\right)-\overline{{\phi\:}_{i}}\right)\text{sin}\left({\phi\:}_{j}\left(t\right)-\overline{{\phi\:}_{j}}\right)}{\sqrt{\sum\limits_{t\epsilon{\tau}_{win}}{\text{sin}}^{2}\left({\phi\:}_{i}\left(t\right)-\overline{{\phi\:}_{i}}\right){\text{sin}}^{2}\left({\phi\:}_{j}\left(t\right)-\overline{{\phi\:}_{j}}\right)}}.$$

Correlations between the upper triangular parts of the matrices yield a dFC matrix. The variance of its upper triangular part defines the fluidity, which is the measure of interest here.

To complement the analysis with a standard sFC analysis, the phase-lag indices (PLI) between full-length time series were computed to define weighted sFC for each subject and frequency band using a code from GitHub [52]. Four basic measures of global topology quantification were adopted from the Brain Connectivity Toolbox [53]: average node strength (*AvNodeStrength*), Louvain modularity (*Modularity*; iterative community finetuning), normalized average clustering coefficient (*NormCluster*), and normalized average characteristic path length (*NormPath*). Normalization was assured by dividing the raw values by averages of values computed for 100 null models of a random network with preserved degree, weight, and strength distributions.

### Between-group differences in functional connectivity

The permutational multivariate analysis of variance (PERMANOVA) [54] was employed using MATLAB 2022a (The MathWorks, Inc., Natick, MA, USA) to test between-group differences in variables with skewed distribution, with age regressed out. The Mahalanobis distance, suitable for dealing with correlated variables, was computed to serve as an input into the PERMANOVA. The significance of the result was drawn from 20,000 permutations. PERMANOVA is robust against bias from non-normal data and unequal variances because it uses permutations rather than distributional assumptions to test group differences in multivariate space.

### Relationship between connectivity and cognitive performance

The Spearman correlation was utilized to evaluate the relationships between dFC and sFC measures (with age regressed out from all variables) on one hand, and performance in three cognitive domains (domains 1–3, see below) and core clinical features [1] of MCI-LB (MDS-UPDRS III, MFS, RBDq, and NPI) on the other hand. The cognitive domains were imputed as latent variables using structural equation modeling (SEM) [55] in IBM AMOS 26.0 software, as described in Supplementary materials, Supplementary Fig. 1 and Supplementary Table 1; and included the domains of (1) executive functions, (2) verbal memory, (3) visuospatial memory, and (4) premorbid intelligence. SEM with latent variables reduces bias from measurement error, overlapping constructs, and shared method variance by modeling the true underlying constructs using multiple observed indicators. Since we were interested in the relationship between connectivity and relative cognitive decline (and not absolute cognitive performance), premorbid intelligence [56] (the fourth domain in the SEM model) was set as a covariate of no interest.

Specifically, Spearman correlations were conducted both in the combined group of all subjects, to represent a continuum from HC to MCI-LB, and separately within group pairs that showed a significant difference in any of the FC measures. Only FC features that significantly differed between the three groups were included in these correlation analyses. Based on prior literature reporting decreased fluidity in aging and states of low consciousness [27, 37], we hypothesized a directional effect of fluidity changes in our study (e.g., reflecting network disruption or compensation). Accordingly, a one-sided FDR correction was applied.

### Association with LC integrity

Firstly, between-group differences in LC integrity were estimated using PERMANOVA with 20,000 iterations, based on Mahalanobis distances of the right caudal LC signal intensity. Secondly, a multiple regression model with an interaction term was conducted in R [57] using the lm function for model estimation. The model investigated the effect of depletion of neuromelanin in the right caudal LC (“rc_LC”) on dFC fluidity (“fcd”), across two levels of premorbid intelligence (“GC_2lev”). Similar analyses were conducted exploratorily for other LC areas; the results are reported in Supplementary results.

*lm(fcd ~ poly(rc_LC*, 2) * GC_2lev).

The second-order polynomial interaction term tested whether the effect of LC integrity is quadratic rather than linear, as shown in previous findings [58–60]. The premorbid intelligence variable was dichotomized into two groups based on the median split of its distribution. This allowed for the exploration of differential effects of LC integrity on fluidity across two premorbid intelligence levels (lower vs. higher). Age was regressed out from all variables. The model accounted for 103 observations on all subjects after excluding cases with missing data. Model fit was evaluated using ordinary least squares (OLS) regression.

### Data Availability

Data is available upon reasonable request.

## Results

### Participants

A total of 126 subjects were enrolled in the study, comprising 58 CN-CCF, 39 with MCI-LB, and 29 HC. The demographics, cognitive domains, core clinical DLB features, and blood biomarker values are presented in Table 1.

**Table 1:** Clinical characteristics

	Median (Q1, Q3) or count			*p* (PERMANOVA or chi-square test)	
	HC	CN-CCF	MCI-LB	CN-CCF vs. HC	MCI-LB vs. HC
N (males/females)	29 (13/16)	58 (22/36)	39 (14/25)	0.536	0.457
Age	68 (62.75, 74.0)	65 (60.0, 69.0)	70 (67.0, 73.0)	0.066	0.461
GFAp	149 (124, 223)	153 (109, 200)	183 (127, 266)	0.938	0.065
NfL -/+	14/11	35/19	16/22	0.453	0.280
pTau181 -/+	24/1	46/8	29/9	0.159	0.074
pTau217 -/+	22/3	47/7	24/14	0.905	(L) 0.030
pTau231 -/+	23/2	50/4	31/7	0.926	0.247
Premorbid intelligence	0.33 (0.17, 0.48)	0.145 (0.02, 0.39)	− 0.08 (-0.30, 0.14)	(L) 0.040	(L) ≤ 0.0001
Executive functions	0.36 (-0.01, 0.44)	0.22 (0.01, 0.35)	− 0.36 (-0.73, − 0.08)	0.367	(L) ≤ 0.0001
Visual memory	0.65 (0.15, 1.01)	0.69 (0.41, 1.05)	− 0.28 (-1.34, 0.18)	0.539	(L) ≤ 0.0001
Verbal memory	0.54 (-0.06, 0.94)	0.7 (0.20, 1.17)	− 0.54 (-1.36, − 0.02)	0.291	(L) ≤ 0.0001
MoCA	27 (26.0, 29.0)	27 (26.0, 28.0)	24 (21.0, 26.0)	0.299	(L) ≤ 0.0001
UPSIT	10 (8.25, 10.75)	9 (8.0, 10.0)	7 (5.5, 10.0)	0.501	(L) 0.0012
MDS-UPDRS III†	0 (0, 1.0)	4 (2.75, 7.0)	6 (3.0, 9.0)	(H) ≤ 0.0001	(H) ≤ 0.0001
ESS	5.5 (3.0, 7.0)	8 (6.0, 12.0)	8 (5.0, 11.0)	(H) ≤ 0.0001	(H) 0.0005
RBDq†	1.5 (0, 3.0)	4 (2.0, 6.0)	4 (2.0, 5.0)	(H) ≤ 0.0001	(H) ≤ 0.0001
GDS	3 (1.0, 6.0)	7 (3.75, 11.0)	8 (4.0, 11.0)	(H) 0.0007	(H) 0.0003
MFS†	0 (0, 0)	1 (0, 1)	1 (0, 2)	(H) ≤ 0.0001	(H) ≤ 0.0001
NPI (0/1) †	29/0	46/10	30/7	(H) 0.015	(H) 0.013

P – p-values where: L – significantly lower in the patient groups than in the healthy controls; H – significantly higher in the patient groups than in the healthy controls. †Scales included in the core clinical criteria for prodromal DLB. NfL – neurofilament light; GFAp – glial fibrillary acidic protein; MoCA – Montreal Cognitive Assessment [61]; UPSIT – University of Pennsylvania Smell Identification Test [62]; MDS-UPDRS III – Movement Disorders Society - Unified Parkinson’s disease rating scale part 3 [63]; ESS – Epworth Sleepiness Scale [64]; RBDq – REM Sleep Behavior Disorder Questionnaire [65]; GDS – Geriatric Depression Scale [66]; NPI – Neuropsychiatric Inventory Questionnaire [i^67^]; MFS – Mayo Fluctuations Scale [68]; HC – cognitively healthy; CN-CCF – cognitively healthy with core symptoms of prodromal Lewy body disease; MCI-LB – mild cognitive impairment with Lewy body disease. NfL and pTau epitopes are reported as negative/positive based on cut-off scores reported in the Methods section

The CN-CCF and MCI-LB groups did not differ significantly from the HC in gender and age. The plasma levels of pTau217 were significantly higher in MCI-LB than in HC; the GFAp and pTau181 levels were higher in MCI-LB than in HC, but this did not reach statistical significance. The MCI-LB group showed significant differences from HC in all cognitive domains and clinical features. The CN-CCF group differed from HC in all clinical features except for smell loss; CN-CCF were similar to HC in terms of cognitive domains and MoCA, except for slightly lower premorbid intelligence. Between-group comparisons of individual cognitive tests included in the cognitive domains are presented in Supplementary Table 2.

**Fig. 1:**
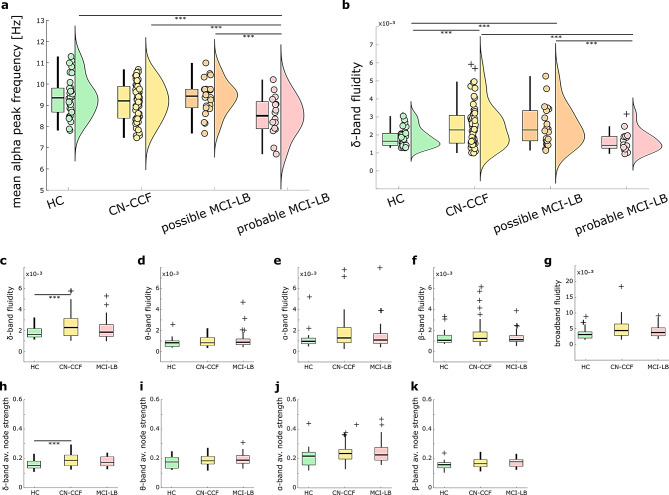
Between-group differences in EEG alpha dominant frequency and δ-band fluidity. **(A)** Mean alpha peak frequency over posterior scalp electrodes. **(B)** δ-band fluidity differences between groups, including MCI-LB subgroups. **C-G)** Fluidity differences across frequency bands. **H-K)** Average node strength differences across frequency bands. Sample sizes: (n_HC_ = 29, n_CN−CCF_ = 58, n_MCI−LB_ = 39 (possible MCI-LB = 22, probable MCI-LB = 16); PERMANOVA, *p* <.05, FDR corrected. *** Significant differences. The boxplots, scatterplots, and half-violin plots were constructed using the daviolinplot function in Matlab [69]. In boxplots, black crosses (+) marks outliers, boxes show median lines, and their borders indicate the 25th and 75th percentiles

We divided the MCI-LB group based on the number of core features into possible MCI-LB (one LB core feature, *n* = 22) and probable MCI-LB (multiple LB core features, *n* = 16) [38]. This subdivision was supported by the characteristic shift of the alpha peak in the power spectrum towards lower frequencies (in sensor space focusing on the electrodes placed on the posterior scalp; the analysis methodology described in Supplementary materials). Pairwise PERMANOVA comparisons (F(3,113) = 5.01, *p* =.005) revealed that the alpha peak significantly differed between HC and probable MCI-LB (F = 10.37, *p* =.003), CN-CCF and probable MCI-LB (F = 6.21, *p* =.020), and possible MCI-LB and probable MCI-LB (F = 8.74, *p* =.006). Figure 1a depicts this effect at *p* <.05 (FDR corrected).

### Transient changes in functional connectivity across groups

We analyzed the topology and dynamics of the FC of the source-reconstructed resting state EEG. Groups HC, CN-CCF, and MCI-LB differed in the average node strength of the sFC across frequency bands (F(2,123) = 1.51; *p* =.023). The post-hoc tests of between-group differences (*p* <.05, two-sided FDR corrected) revealed a significantly higher average node strength in the δ-band (F = 16.38, *p* =.0003) in CN-CCF compared to HC. This hyperconnectivity diminishes with progression to the MCI stage; the results for all studied FC features are reported in detail in Supplementary Table 3. Our exploratory analyses further indicated a more random topology, captured by diverse network properties (lower modularity, normalized average clustering coefficient, and normalized average characteristic path length), across frequency bands in patient groups compared to HC.

The HC, CN-CCF, and MCI-LB groups also differed in the age-regressed temporal variability (fluidity) of the dFC across frequency bands (F(2,123) = 1.86, *p* =.037), reflecting differences in recurring co-activation states and the transitions between highly defined and diffused states. The post-hoc tests of between-group frequency-specific differences (*p* <.05, two-sided FDR corrected) revealed higher δ-band fluidity in CN-CCF compared to HC (F = 12.72, *p* =.001). All the results are summarized in Supplementary Table 4.

Since our hypothesis that fluidity lowers with clinical severity was not supported, we conducted an exploratory analysis to examine the differences in fluidity between possible MCI-LB and probable MCI-LB subjects. Significantly higher δ-band fluidity was observed in CN-CCF and in possible MCI-LB as compared to HC; in higher stages of clinical severity, δ-band fluidity was significantly lower in probable MCI-LB as compared to CN-CCF and possible MCI-LB (*p* <.05, two-sided FDR corrected). Figure 1b captures these results, accompanied by Supplementary Table 5.

The results reported here for sFC and dFC, controlling for the effect of age, also hold when controlling for the effects of both age and sex. All results are summarized in Supplementary Tables 3b for sFC, 4b for dFC, and 5b for the subdivision of the MCI-LB group into possible and probable MCI-LB.

### Association between functional connectivity and clinical profile

**Fig. 2:**
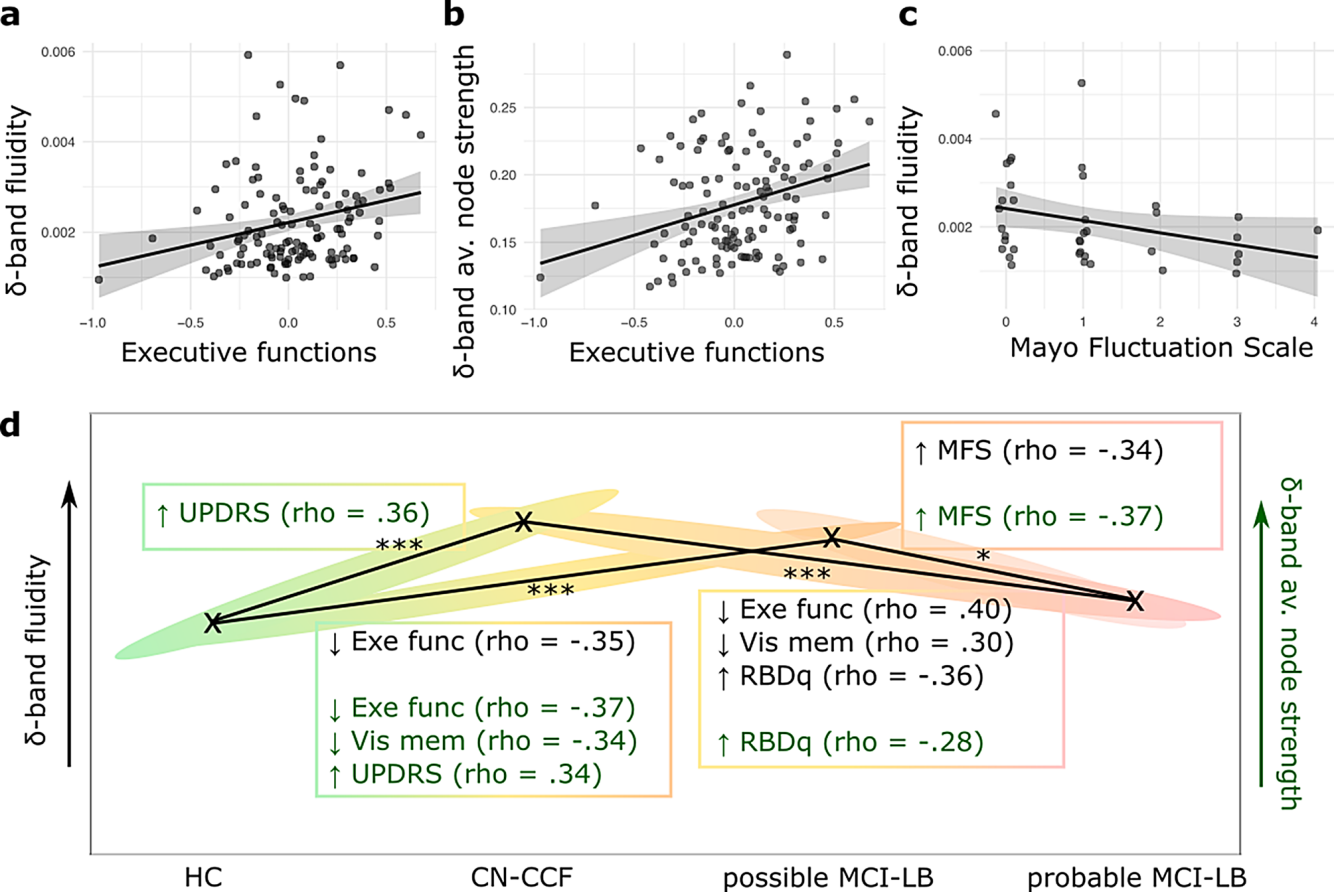
Relationships between EEG features, cognition, and core clinical features of prodromal DLB. Scatterplots depict significant partial Spearman correlations, with premorbid intelligence and age regressed out, between **(a)** executive functions (computed as a composite latent variable from SEM) and δ-band fluidity in all subjects (rho = 0.26, *p* =.004), **(b)** executive functions and δ-band average node strength in all subjects (rho = 0.28, *p* =.002), **(c)** Mayo Fluctuation Scale and δ-band fluidity in MCI-LB (rho = − 0.34, *p* =.034); note that a higher MFS score indicates greater impairment. Shaded ribbons represent ± 1 standard error. **(d)** Graph showing the significant (*p* <.05, one-sided FDR corrected) relationships between EEG markers, cognition, and core clinical features of prodromal DLB displayed across group pairs, where significant (*p* <.05, FDR corrected) dFC and sFC differences were identified (n_HC_ = 29, n_CN−CCF_ = 58, n_possible MCI−LB_ = 22, n_probable MCI−LB_ = 16)

Following our second hypothesis, we evaluated the relationship between dFC fluidity and cognitive decline while grouping all subjects together. After regressing out the effect of premorbid intelligence, fluidity and average node strength were positively correlated with executive functions (rho = 0.26, *p* =.004; rho = 0.28, *p* =.002, respectively; Fig. 2a and b); these associations were not significant for other cognitive domains or when premorbid intelligence was not accounted for. Detailed results are available in Supplementary Table 6.

Furthermore, Spearman correlations were conducted in significant group pairs to capture group-specific relationships between the δ-band average node strength (observed as higher in CN-CCF compared to HC) and δ-band fluidity (higher in the CN-CCF and possible MCI-LB groups as compared to HC) on one side and core clinical features and cognitive domains on the other side. We found that the significant increase in δ-band average node strength in the CN-CCF group compared to HC was positively correlated with motor symptoms (UPDRS score; rho = 0.362, *p* =.001). This positive relationship also held when comparing HC to the possible MCI-LB group (rho = 0.337, *p* =.016). Additionally, higher δ-band average node strength was significantly associated with lower executive function performance (rho =–0.369, *p* =.008) and poorer visual memory (rho =–0.340, *p* =.016). A similar negative correlation with executive function was also observed for δ-band fluidity (rho =–0.349, *p* =.012). As fluidity decreased further—particularly along the trajectory from CN-CCF to probable MCI-LB, and from possible to probable MCI-LB—the direction of these relationships changed. Specifically, we found significant positive correlations between δ-band fluidity and executive functions (rho = 0.279, *p* =.016), as well as with visual memory (rho = 0.273, *p* =.019). In contrast, both δ-band average node strength and δ-band fluidity were negatively correlated with RBD questionnaire scores (rho =–0.361, *p* =.002 and rho =–0.284, *p* =.016, respectively). Finally, within the MCI-LB group, as hypothesized, δ-band fluidity and δ-band average node strength were negatively correlated with clinical fluctuations (MFS; rho =–0.34; *p* =.034 and rho =–0.37; *p* =.022, respectively; uncorrected; see Fig. 2c). Spearman correlations with cognitive tests and scales in group pairs are summarized in Fig. 2d, and in Supplementary Table 7.

Additional exploratory analyses examining correlations between plasma biomarkers and the FC features that showed significant group differences were conducted. When considering all participants together, no statistically significant associations were observed. These results are summarized in Supplementary Table 8.

### Correlation between the fluidity and structural integrity of the locus coeruleus

**Fig. 3:**
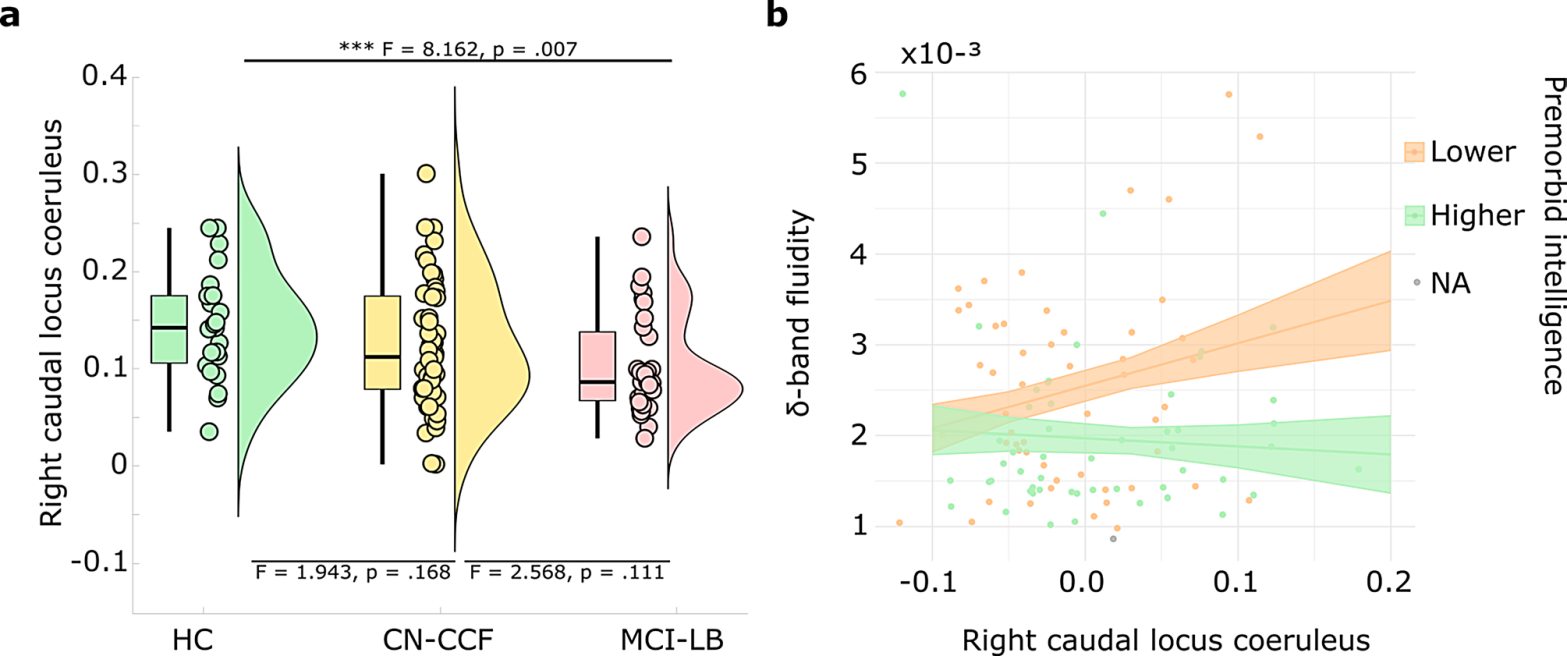
Interactions: right caudal locus coeruleus and premorbid intelligence. **(a)** Between-group differences in right caudal locus coeruleus signal intensity (n_HC_ = 23, n_CN−CCF_ = 49, n_MCI−LB_ = 32). **(b)** Locus coeruleus signal intensity significantly predicted δ-band fluidity only in subjects with lower premorbid intelligence; premorbid intelligence subgroups consisted of n_lower_ = 52, n_higher_ = 51. Age was regressed out from all variables. Shaded ribbons represent ± 1 standard error

Firstly, the three groups (HC, CN-CCF, and MCI-LB) significantly differed in the right caudal LC signal intensity (F = 3.635, *p* =.032). Post-hoc tests showed significantly lower values in the MCI-LB group compared to HC (*p* <.05, two-sided FDR corrected); see Fig. 3a.

To examine how the disruption of the neuromodulatory system relates to the dFC changes, we assessed the relationship between the depletion of neuromelanin in LC and fluidity using a linear regression model across the two levels of premorbid intelligence. Our model (F(5,97) = 3.56, *p* =.005) shows that depletion of neuromelanin in the right caudal LC is associated with lower δ-band fluidity, particularly in subjects with lower premorbid intelligence (β = − 0.005, *p* =.014; see Fig. 3b and Supplementary Table 9). Supplementary Table 10 presents analogous exploratory models with other LC regions – caudal left and middle, rostral right and left, where only the main effect of the left caudal region in predicting δ-band fluidity was observed without the interaction with premorbid intelligence.

## Discussion

The present study combined resting state EEG and neuromelanin MRI to examine the relationship between symptom severity, cognitive decline, and changes in the spatio-temporal characteristics of FC, while also evaluating the contribution of the LC neuromodulatory system to early FC changes in very early pDLB stages.

First, we hypothesized a linear decline in fluidity and static connectivity measures; however, we observed a nonlinear trajectory, specifically in δ-band fluidity and average node strength, from HC to probable MCI-LB. This was supported by significant group differences where the groups with intermediate symptom severity (CN-CCF and possible MCI-LB) exhibited higher connectivity measures as well as a negative association with cognition and a positive association with increased motor symptoms of parkinsonism. Correlations in the opposite direction were observed between cognitive and EEG outcomes between the two intermediate groups and the probable MCI-LB group. The significant increase from HC to CN-CCF suggests elevated cognitive compensation as the two groups did not differ cognitively, but CN-CCF already displayed some core clinical features of pDLB, such as motor symptoms of parkinsonism, fluctuating alertness, REM sleep behavioral disorder, and hallucinations. Fluidity changing its course and being higher in the CN-CCF group than in the probable MCI-LB group, coupled with objectively decreasing cognition to mild cognitive decline, is consistent with the notion that less variable brain dFC is related to worsening of cognitive performance [27]. Employing a different approach to assess fluctuations of brain activity in the resting state, Schumacher et al. [28] observed generalized slowing in temporal dynamics of electrophysiological spatial states in individuals with progressed DLB, who spent more time in a single state than healthy controls. Correspondingly, we observed a decrease in delta-fluidity from CN-CCF to probable MCI-LB, and we may speculate that this decrease continues with disease progression.

Considering the relationship between the dFC fluidity and the cognitive performance alone, we show that fluidity was associated with executive functions across the whole spectrum of HC–MCI-LB (after accounting for premorbid intelligence as a proxy of cognitive reserve). The association with the fluctuations in the alertness-activation dimension (i.e., daytime drowsiness and feeling lethargic, daytime sleepiness, evaluated here by MFS) was observed only in the cognitively impaired – the MCI-LB group. This further supports the notion that the increased fluidity in the CN-CCF group may be linked to different—potentially compensatory—mechanisms. This is also in line with Schumacher et al. [28] who observed that EEG spatial states were more strongly related to cognitive-attention dimension of fluctuations and less to the alertness-activation dimension.

Static FC exhibited a similar non-linear trend, with increased δ-band average node strength from HC to CN-CCF, followed by a significant weakening when progressing into MCI-LB. This finding may point to early hyperconnectivity, which was reported as an early neurodegeneration phase response [35]. Studies in PD have consistently shown that the correlation between the loss of dopaminergic cells and the severity of clinical symptoms is moderate [70–72], which aligns with our results indicating that the relationship between brain pathology and clinical manifestation may be partially mitigated by other compensatory mechanisms, including dFC fluidity or global hyperconnectivity.

**Fig. 4:**
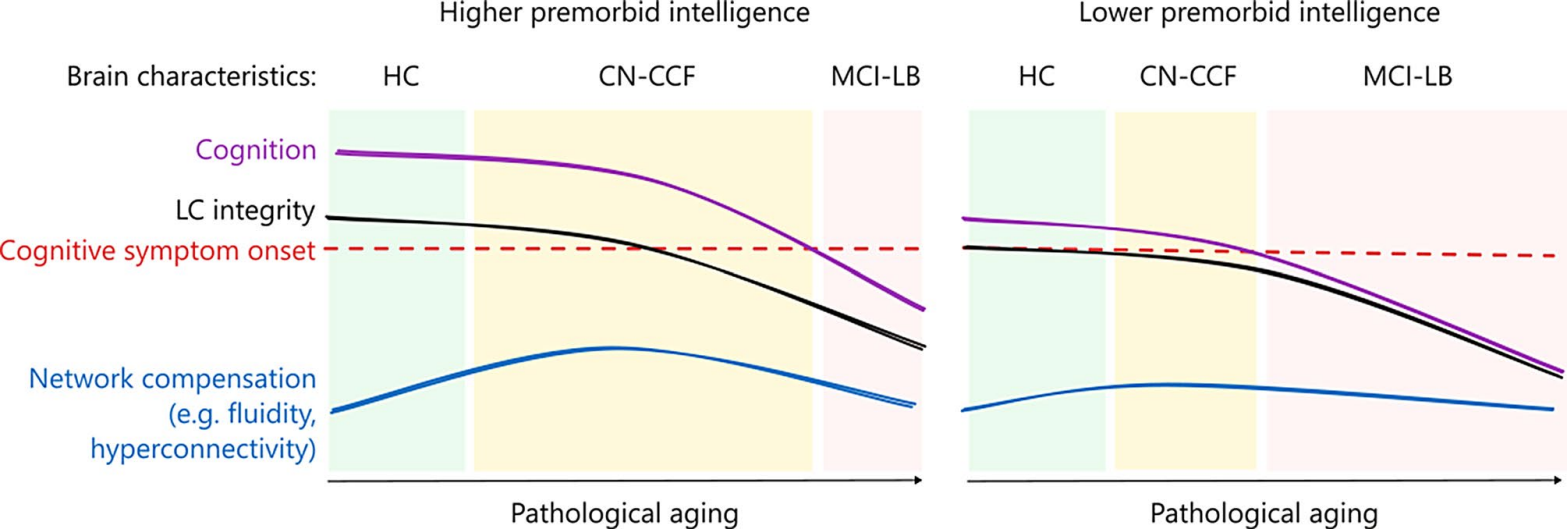
Overview of the main findings of this study in the context of aging and lifespan studies. A theoretical model showing cognitive trajectories over the life course from late adulthood into the prodromal phase of neurodegenerative diseases. The figure depicts two hypothetical trajectories of individuals with higher and lower premorbid intelligence. The cognitive trajectories have different intercepts, representing initially higher and lower levels of cognitive ability, respectively, which are strongly determined by education [33, 34]. Throughout aging, the levels of cognitive ability are shaped by brain health (including locus coeruleus integrity) [26, 58, 74] and eventually descend toward a dysfunctional threshold at advanced chronological ages. Although environmental enrichment, such as education, has a negligible impact on the rate of cognitive decline [33, 34], our present work, in line with others, suggests that declining brain health is compensated by various mechanisms. These include functional network hyperconnectivity [35] and fluidity, which have been reported to be higher in individuals with better cognitive functioning [18, 19] and to follow nonlinear changes, such as transient increase during the cognitively less intact prodromal phase, followed by a slowing in MCI [13, 14]

In a brain network modeling study, Lavanga et al. [27] showed that the age-related decrease in brain fluidity is associated with increased modulation of brain dynamics by structural connectivity and reduced cognitive performance [29, 30]. Notably, this relationship was particularly stronger in low-performing individuals. Our findings, supported by both FC modalities, complement the established relationship between brain fluidity and cognition in healthy aging and neurodegenerative disease, and expand it to the early prodromal phase of a neurodegenerative disease, where the brain appears to leverage dynamic reconfiguration and heightened connectivity to maintain cognitive performance. However, as neurodegeneration progresses, these mechanisms become weaker or even maladaptive [73]. We complement this interpretation with a graph in Fig. 4.

Similarly, our results for δ-band static FC align with δ-band connectivity changes reported during healthy aging [75]. The authors show a gradual decrease in δ-band connectivity from youth to middle age, with a further decrease specifically localized within regions involved in memory and visual processing in late adulthood. They also observed an accompanying diffuse δ-band hyperconnectivity in older adults, interpreted as a consequence of broader network disruption. Another pattern of age-related disruption has also been demonstrated in δ-band connectivity, represented by the increase in the normalized characteristic path length [76]. Studies comparing healthy adults with individuals diagnosed with MCI, Alzheimer’s disease, or vascular dementia [77–81] consistently reported a decrease in δ-band connectivity as a marker of the diseases.

Finally, our findings supported the hypothesis that early changes in fluidity are associated with the right caudal LC integrity, which has been shown to deteriorate in MCI-LB [23]. This aligns with Matar et al. [5], who found that regional alterations in the dynamic brain topology in DLB correlate with the distribution pattern of cholinergic and adrenergic receptors, key neuromodulators supporting attention and wakefulness, contributing to the DLB-characteristic fluctuations. In our study, this relationship between LC and fluidity was evident only in individuals with estimated lower premorbid intelligence, suggesting that those with higher premorbid intelligence may compensate for LC deterioration through other mechanisms. This is consistent with cognitive reserve research, in which social activity buffered the effect of LC neurofibrillary tangle density pathology on cognitive decline in elderly subjects [82]. Replicating this result in individuals at the prodromal stage of DLB suggests that the moderating effect of cognitive reserve on the relationship between the noradrenergic system and cognitive performance seems to be similar across both healthy and pathological aging trajectories. Furthermore, we argue that the elevated fluidity and node strength in the CN-CCF represent compensation, given the preserved cognitive performance in CN-CCF. Alternatively, in the presence of neuropathology, neural activity may initially increase as a compensatory response until a limit is reached [83]; further increases may reflect toxic overactivation, decline, and maladaptation [84]. However, disentangling the role of compensatory mechanisms along individual trajectories would greatly benefit from longitudinal data. Our results present an entry point for employing mechanistic brain modeling [85], with the potential to further elucidate the causal relationships between structural changes and observed functional data features across the individual trajectories.

Cognitive fluctuations in DLB manifest on multiple timescales, ranging from seconds, minutes, and hours to days, weeks, and even months [6]. At the short timescale, changes in EEG provide evidence of physiological correlates of these fluctuations [28, 86]. Abnormal switching between normal cortical desynchronization (arousal) and increased synchronization (drop in attention or arousal) may be reflected in transient changes in low-frequency power and connectivity. These dynamics alter the variability of functional connectivity, which is captured in our analyses by the fluidity measure. At the cellular and molecular level, disruptions in key neuromodulatory systems have been implicated in the emergence of fluctuations [5]. In our data, we were able to relate functional observations to reduced structural integrity of the LC (noradrenergic system). Moreover, cholinergic system impairment probably precedes structural degeneration [87], as the alpha-synuclein has been shown to reduce cholinergic neurotransmitter production [88].

By describing both neurodegenerative and compensatory (or maladaptive) processes in prodromal DLB, our findings contribute to the development of staging models that capture this dynamic interplay. Notably, fluidity and LC integrity emerge as promising candidate biomarkers for early patient stratification, helping to identify individuals at greater risk of progression. Their sensitivity to both pathological and compensatory processes further underscores their utility for therapeutic monitoring and as inclusion criteria in clinical trials targeting early disease stages.

Our study has the following limitations. First, the absence of DaT SPECT imaging as an indicative biomarker. Nonetheless, its sensitivity in detecting MCI-LB is relatively low, with estimates around 66% [38, 89]. The seed amplification assay for detecting pathological α-synuclein in CSF or skin is not yet available in our country, even for research use. The second limitation is the use of the MFS to assess cognitive fluctuations and the NPI to assess the presence of hallucinations. Although both scales are clinically validated and useful for screening, they include only four and two items, respectively, which may not fully capture the multidimensional nature or temporal dynamics of attentional fluctuations and hallucinations. Replicating these findings using more fine-grained behavioral or physiological measures—such as continuous performance tasks for cognitive fluctuations—would enhance the precision and interpretability of the results. The third limitation concerns the sample size. Although the total sample of 126 participants is relatively large for a multimodal imaging study, stratifying the MCI-LB group into subgroups led to smaller sample sizes, which limits the generalizability of subgroup-specific findings. Replication in larger, independent cohorts is therefore warranted.

Overall, this study advances the understanding of the neurochemical bases of neurophysiological alterations in prodromal DLB patients, of which noradrenergic mechanisms remain particularly underexplored. Future research with a longitudinal study design will be needed to substantiate the causal nature of these relationships.

## Electronic supplementary material

Below is the link to the electronic supplementary material.

Supplementary Material 1

## Data Availability

Data are available upon reasonable request.

## Abbreviations

AAL: Automated anatomical atlas
CCF: Core clinical features
CCor: Circular correlation coefficient
CN: CCF-Cognitively normal subjects with core clinical features
CR: Contrast ratio
dFC: Dynamic functional connectivity
DLB: Dementia with Lewy bodies
ESS: Epworth Sleepiness Scale
FC: Functional connectivity
fcd: Fluidity
GC_2lev: Two levels of premorbid intelligence
GDS: Geriatric Depression Scale
GFAp: Glial fibrillary acidic protein
HC: Healthy controls
LC: Locus coeruleus
LORETA: Low-resolution electromagnetic tomography analysis
LSMAC: Locally Spherical Model with Anatomical Constraints
MCI: Mild cognitive impairment
MCI-LB: Subjects with mild cognitive impairment with Lewy bodies
MDS-UPDRS III: Unified Parkinson’s Disease Rating Scale, part 3
MFS: Mayo Fluctuations Scale
MNI: Montreal Neurological Institute
MoCA: Montreal Cognitive Assessment
NfL: Neurofilament light chain
NMS: MRI-Neuromelanin-sensitive MRI
NPI: Neuropsychiatric Inventory questionnaire
OLS: Ordinary least squares
pDLB: prodromal DLB
PERMANOVA: Permutational multivariate analysis of variance
PLI: Phase-lag index
PT: Pontine tegmentum
RBDq: REM Sleep Behavior Disorder questionnaire
rc_LC: Right caudal locus coeruleus
ROC: Receiver operating characteristic
SEM: Structural equation modeling
sFC: Static functional connectivity
UPSIT: University of Pennsylvania Smell Identification Test
