# Supplementary material for: Early functional changes in lewy body dementia: roles of dynamics, locus coeruleus, and compensation
Source: Alzheimers Res Ther. 2025 Aug 23;17:199. doi: 10.1186/s13195-025-01828-1 (PMC12374355; doi:10.1186/s13195-025-01828-1)
Supplement: Supplementary file 1 — Supplementary Material 1 [file 13195_2025_1828_MOESM1_ESM.docx]

# **Early Functional Changes in Lewy Body Dementia: Roles of Dynamics, Locus Coeruleus, and Compensation**

Kristína Mitterová, Eva Výtvarová, Anežka Kovářová, Martin Lamoš, Jan Fousek, Irena Rektorová

# **Supplementary Methods**

## Latent modeling

We utilized the AMOS software version 26.0 to extract four latent scores of executive functions, verbal and visuospatial memory, and premorbid cognitive abilities in structural equation modeling (SEM) with maximum likelihood estimation. This offers a multivariate approach in which manifest variables can be used to infer unobserved (latent) scores that account for measurement error in observed scores and thereby increase statistical power^1^. Estimation of latent scores over averaged composites accounts for complex dependencies between tests and factors, which is more appropriate in highly intercorrelated psychological data. We used a test of crystallized intelligence and education as estimates of premorbid cognitive abilities, as they are age invariant^2^; all tests that were used for calculations of the latent scores are included in the SEM diagram in Supplementary Figure 1, and model fit is reported in Supplementary Table 1.


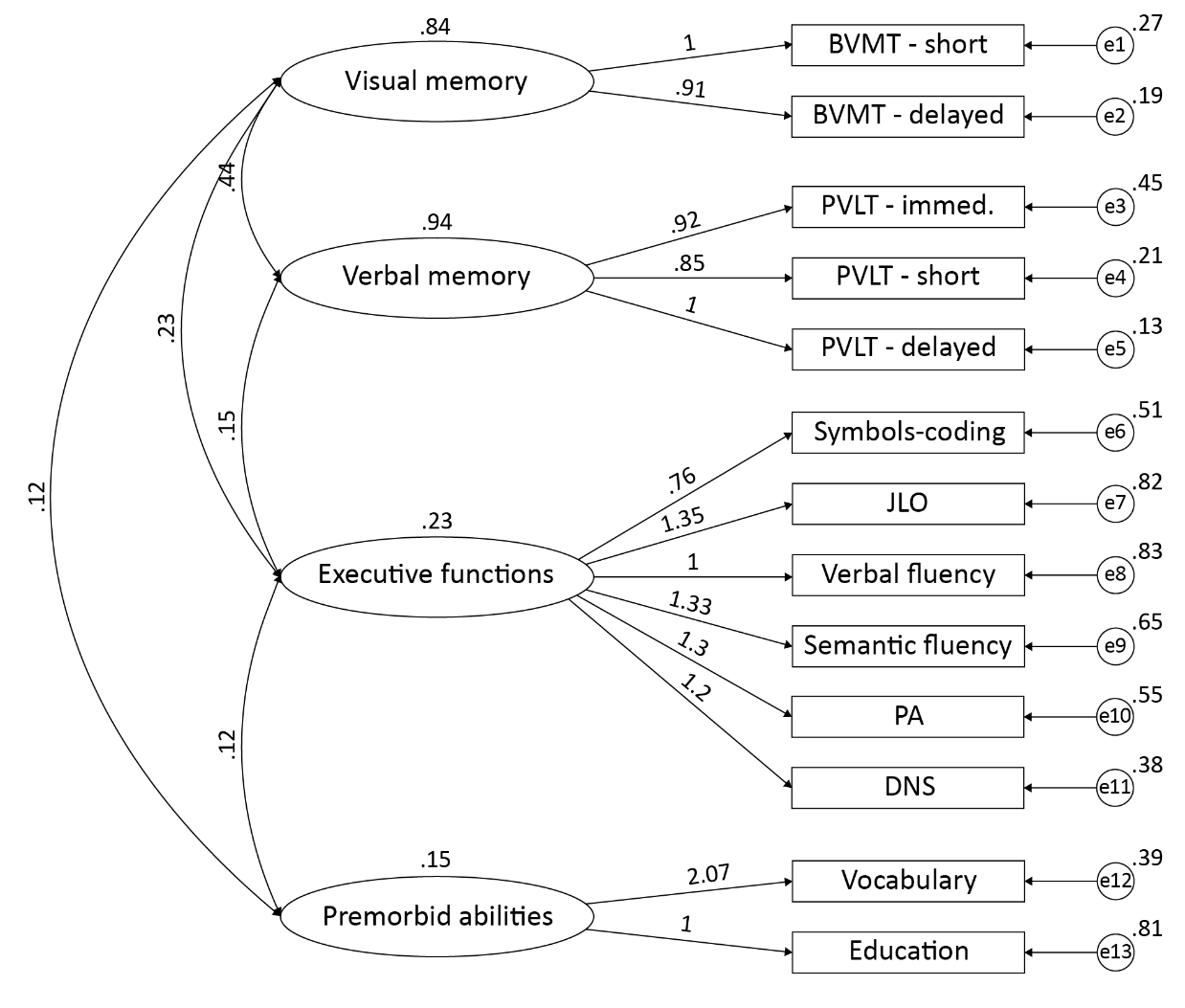


**Supplementary Figure 1: Standardized diagram from the structural equation modeling.** All regression weights between latent and manifest variables are significant on p < .001 except Vocabulary on p = .004. All covariances between latent variables are significant on p < .05. Manifest variables are tests' Z-scores. JLO – Judgement of Line Orientation, PA – Picture Arrangement, BVMT – Brief Visuospatial memory Test, PVLT – Pennsylvania Verbal Learning Test, DNS – Digit Number Sequencing, immed. – immediate recall, short – short-term memory score, delayed – recall after 30 minutes, e – error term.

**Supplementary Table 1: Adequacy of the SEM model.**

| **Fit index** |  |
| --- | --- |
| Chi-square | *χ2*(48) = 87.42, *p*= .012 |
| Root mean square error of approximation RMSEA | .060 |
| TLI rho | .946 |
| The comparative fit index CFI | .959 |

## Alpha power analysis

Electrodes placed on the posterior scalp (PO7, PO3, O1, POz, Oz, PO4, O2, PO8) were chosen to assess the predominant frequency of the alpha peak. The data underwent transformation into the spectral domain using Fast Fourier Transform within a 60s-time frame. The squared absolute values of the Fourier coefficients were normalized, and the local maximum was identified within the spectral range of 7-12 Hz. Subsequently, the median and variance were calculated.

# **Supplementary Results**

**Supplementary Table 2:** **The group differences in individual tests (age-normalized Z-scores) of the neuropsychological battery.** PERMANOVA; D (orange color): decrease (p < .05) in the second group compared to the first group; I (green color): increase (p < .05).

|  | median HC | median CCF | median MCI-LB | CCF from HC | MCI-LB from HC |
| --- | --- | --- | --- | --- | --- |
| JLO | 0.5828 | 0.151 | -1.056 | p = .154 | D, p ≤ .0001 |
| BVMT - short | 0.7 | 0.8 | -0.5 | p = .305 | D, p ≤ .0001 |
| BVMT - delayed | 0.8 | 0.7 | 0 | p = .832 | D, p ≤ .0001 |
| PVLT immediate | 0.5287 | 0.9971 | -0.6248 | p = .207 | D, p ≤ .0001 |
| PVLT short | 0.5378 | 0.5713 | -0.2989 | p = .913 | D, p ≤ .0001 |
| PVLT long delayed | 0.4312 | 0.751 | -0.6913 | p = .230 | D, p ≤ .0001 |
| Symbols-coding | 0.333 | 0 | -0.333 | p = .206 | D, p = .0122 |
| Digit-number seq. | 0.6667 | 0.1667 | -0.5 | p = .168 | D, p ≤ .0001 |
| Vocabulary | 1.000 | 0.500 | 0.000 | p = .072 | D, p ≤ .0002 |
| Verbal fluency lexical | 0.75 | 0.5 | 0 | p = .524 | D, p = .021 |
| Verbal fluency sem. | 0 | 0.5 | -0.25 | p = .069 | D, p = .010 |
| Picture arrangement | -0.1667 | 0 | -1.333 | p = .841 | D, p ≤ .0001 |

**Supplementary Table 3: SFC differences (p-values) across frequency bands, corrected for the effect of age**. PERMANOVA. D (orange color): decrease (p < .05) in the second group compared to the first group, I (green color): increase (p < .05). *** Significant result after FDR correction as reported in the manuscript.

| HC x CN-CCF | AvNodeStrength | Modularity | NormCluster | NormPath |
| --- | --- | --- | --- | --- |
| δ-band | **I,** **.0003***** | D, .040 | D, .003 | .649 |
| θ-band | .076 | .072 | .156 | D, .023 |
| α-band | .058 | D, .026 | .052 | .126 |
| β-band | .072 | D, .042 | D, .014 | .363 |
| HC x MCI-LB | AvNodeStrength | Modularity | NormCluster | NormPath |
| δ-band | I, .022 | .298 | .928 | .98 |
| θ-band | I, .027 | D, .011 | .276 | D, .016 |
| α-band | I, .056 | D, .047 | .190 | D, .019 |
| β-band | I, .027 | D, .030 | .645 | D, .055 |
| CN-CCF x MCI-LB | AvNodeStrength | Modularity | NormCluster | NormPath |
| δ-band | .170 | .377 | I, .014 | .818 |
| θ-band | .576 | .399 | .748 | .607 |
| α-band | .917 | .453 | .361 | .353 |
| β-band | .638 | .788 | .090 | .206 |

**Supplementary Table 3b: SFC differences (p-values) across frequency bands, corrected for the effects of age and sex.** PERMANOVA. D (orange color): decrease (p < .05) in the second group compared to the first group, I (green color): increase (p < .05). *** Significant result after FDR correction. Groups HC, CN-CCF, and MCI-LB differed in global sFC topology across frequency bands (F = 1.51, p = .024).

| HC x CN-CCF | AvNodeStrength | Modularity | NormCluster | NormPath |
| --- | --- | --- | --- | --- |
| δ-band | **I, .0001***** | D, .016 | D, .003 | .517 |
| θ-band | I, .035 | D, .048 | .286 | D, .015 |
| α-band | I, 054 | D, .040 | .058 | .153 |
| β-band | I, .026 | D, .015 | D, .009 | .325 |
| HC x MCI-LB | AvNodeStrength | Modularity | NormCluster | NormPath |
| δ-band | I, .033 | 0.309 | .956 | .955 |
| θ-band | I, .035 | D, .015 | .360 | D, .028 |
| α-band | .114 | D, .065 | .170 | D, .022 |
| β-band | I, .083 | D, .071 | .757 | D, .011 |
| CN-CCF x MCI-LB | AvNodeStrength | Modularity | NormCluster | NormPath |
| δ-band | D, .030 | .142 | I, .003 | .492 |
| θ-band | .869 | .512 | .794 | .918 |
| α-band | .941 | .720 | .554 | .255 |
| β-band | .676 | .620 | I, .076 | .379 |

**Supplementary Table 4: DFC fluidity differences (p-values) across frequency bands, corrected for the effect of age.** PERMANOVA. D (orange color): decrease (p < .05) in the second group compared to the first group, I (green color): increase (p < .05). *** Significant result after FDR correction as reported in the manuscript.

|  | HC x CN-CCF | HC x MCI-LB | CN-CCF x MCI-LB |
| --- | --- | --- | --- |
| δ-band | **I, .0013***** | I, .068 | .142 |
| θ-band | .196 | .157 | .585 |
| α-band | .199 | .413 | .947 |
| β-band | I, .085 | .956 | D, .051 |
| broadband | I, .005 | I, .089 | .191 |

**Supplementary Table 4b: DFC fluidity differences (p-values) across frequency bands, corrected for the effects of age and sex.** PERMANOVA. D (orange color): decrease (p < .05) in the second group compared to the first group, I (green color): increase (p < .05). *** Significant result after FDR correction. Groups HC, CN-CCF, and MCI-LB also differed in dFC fluidity across frequency bands (F = 1.79, p = .047).

|  | HC x CN-CCF | HC x MCI-LB | CN-CCF x MCI-LB |
| --- | --- | --- | --- |
| δ-band | **I, .0014***** | I, .069 | .142 |
| θ-band | .266 | .225 | .609 |
| α-band | .246 | .493 | .970 |
| β-band | I, .089 | .905 | D, .052 |
| broadband | I, .006 | I, .101 | .191 |

**Supplementary Table 5: DFC fluidity differences (p-values) in δ-band, corrected for the effect of age.** PERMANOVA. D (orange color): decrease (p < .05) in the second group compared to the first group, I (green color): increase (p < .05). *** Significant result after FDR correction as reported in the manuscript.

|  | δ-band fluidity |
| --- | --- |
| HC x CN-CCF | **I, .0013***** |
| HC x MCI-LB possible | I, .007*** |
| HC x MCI-LB probable | .496 |
| CN-CCF x MCI-LB possible | .909 |
| CN-CCF x MCI-LB probable | **D, .002***** |
| MCI-LB-possible x MCI-LB probable | **D, .003***** |

**Supplementary Table 5b:** **DFC fluidity differences (p-values) in δ-band, corrected for the effects of age and sex.** PERMANOVA. D (orange color): decrease (p < .05) in the second group compared to the first group, I (green color): increase (p < .05). *** Significant result after FDR correction.

|  | δ-band fluidity |
| --- | --- |
| HC x CN-CCF | **I, .0014***** |
| HC x MCI-LB possible | **I, .007***** |
| HC x MCI-LB probable | .455 |
| CN-CCF x MCI-LB possible | .912 |
| CN-CCF x MCI-LB probable | **D, .002***** |
| MCI-LB-possible x MCI-LB probable | **D, .004***** |

**Supplementary Table 6: Spearman correlations across all subjects between FC and clinical profile, corrected for the effect of age.** Specifically, δ-band average node strength and δ-band fluidity on the one hand, and cognitive domains and LBD core clinical features on the other hand. Orange color: negative correlation, green color: positive correlation. *** Significant result after FDR correction as reported in the manuscript.

|  | Full Spearman correlation | | Partial corr. covar: Premorbid intelligence | |
| --- | --- | --- | --- | --- |
|  | δ-band fluidity | δ-band AvNodeStrength | δ-band fluidity | δ-band AvNodeStrength |
| Premorbid intelligence | p = .155  rho = -.127 | p = .026  rho = -.198 | NaN  - | NaN  - |
| Executive functions | p = .707  rho = .034 | p = .874  rho = -.014 | **p = .004*****  **rho = .263** | **p = .002*****  **rho = .276** |
| Verbal memory | p = .459  rho = .067 | p = .167  rho = .124 | p = .277  rho = .098 | p = .050  rho = .176 |
| Visual memory | p = .579  rho = .050 | p = .863  rho = .016 | p = .133  rho = .135 | p = .122  rho = .139 |
| UPDRS | p = .066 rho = .166 | p = .008  rho = .24 | p = .262 rho = .103 | p = .111 rho = .145 |
| MFS | p = .443 rho = -.070 | p = .957  rho = -.005 | p = .215 rho = -.114 | p = .499 rho = -.062 |
| RBDq | p = .306 rho = -.092 | p = .837  rho = -.019 | p = .194 rho = -.118 | p = .612 rho = -.046 |
| NPI | p = .580  rho = -.050 | p = .705  rho = -.035 | p = .520  rho = -.059 | p = .414  rho = -.075 |

**Supplementary Table 7: Group-specific Spearman correlations between FC and clinical profile, corrected for the effect of age**. Orange color: negative correlation, green color: positive correlation. *** Significant result after FDR correction as reported in the manuscript. ** Significant uncorrected results as reported in the manuscript.

|  | HC+CN-CCF | | HC+ possible MCI-LB | | CN-CCF+ probable MCI-LB | | MCI-LB | |
| --- | --- | --- | --- | --- | --- | --- | --- | --- |
| **p-value** | δ-band fluidity | δ-band Av Node Strength | δ-band fluidity | δ-band Av Node Strength | δ-band fluidity | δ-band Av Node Strength | δ-band fluidity | δ-band Av Node Strength |
| Exe func | .893 | .897 | .012*** | .008*** | .016*** | .086 | .373 | .643 |
| Verb mem | .549 | .245 | .431 | .459 | .339 | .093 | .484 | .179 |
| Vis mem | .921 | .750 | .026 | .016*** | .019*** | .044 | .725 | .516 |
| UPDRS | .006 | .001*** | .103 | .016*** | .306 | .233 | .4525 | .734 |
| MFS | .555 | .152 | .697 | .947 | .089 | .342 | .034** | .022** |
| RBDq | .990 | .407 | .082 | .165 | .002*** | .016*** | .035 | .035 |
| NPI | .487 | .659 | .218 | .322 | .211 | .275 | .909 | .909 |
| **rho** |  |  |  |  |  |  |  |  |
| Exe func | .015 | -.014 | -.349 | -.369 | .279 | .201 | .147 | .077 |
| Verb mem | .065 | .126 | -.113 | -.106 | .113 | .197 | .115 | .220 |
| Vis mem | .011 | -.035 | -.311 | -.340 | .273 | .235 | .058 | .107 |
| UPDRS | .294 | .362 | .231 | .337 | .122 | .143 | -.126 | -.057 |
| MFS | .065 | .157 | .055 | .009 | -.205 | -.115 | -.344 | -.370 |
| RBDq | -.001 | .090 | .244 | .196 | -.361 | -.284 | -.343 | -.343 |
| NPI | -.076 | .048 | .176 | .142 | -.150 | -.131 | .019 | -.019 |

**Supplementary Table 8: Spearman correlations across all subjects between FC and plasma biomarkers, corrected for the effect of age.** Specifically, δ-band average node strength and δ-band fluidity were correlated with levels of GFAp, NfL, pTau181, pTau217, and pTau231. The results are consistent when regressing out the effect of premorbid intelligence.

| GFAp x δ-band fluidity / av. node strength | NfL x δ-band fluidity / av. node strength | pTau181 x δ-band fluidity / av. node strength | pTau217 x δ-band fluidity / av. node strength | pTau231 x δ-band fluidity / av. node strength |
| --- | --- | --- | --- | --- |
| p = .522 / .702  rho = -.060 / -.036 | p = .231 / .389  rho = .112 / .081 | p = .559 / .529  rho = .055 / .059 | p = .200 / .070  rho = .138 / .194 | p = .768 / .938  rho = .028 / .007 |

**Supplementary Table 9:** Linear regression model predicting δ-band fluidity from right caudal LC signal intensity and premorbid intelligence.

| Predictor | Estimate (B) | SE | t | p |
| --- | --- | --- | --- | --- |
| Intercept | .003 | .000 | 18.577 | < .001 |
| Linear: rc LC | .004 | .002 | 2.513 | .014 |
| Quadratic: rc LC | .003 | .002 | 1.821 | .072 |
| Premorbid intelligence: High | -.001 | .000 | -3.237 | .002 |
| Linear: rc LC × Premorbid intelligence: High | -.005 | .002 | -2.512 | .014 |
| Quadratic: rc LC × Premorbid intelligence: High | -.001 | .002 | -.554 | .581 |

Note: rc LC – right caudal locus coeruleus. Estimates reflect unstandardized coefficients. SE = standard error. Premorbid intelligence: Low is the reference group

**Supplementary Table 10**: Exploratory linear regression models predicting δ-band fluidity from LC signal intensity and premorbid intelligence.

| **Predictor** | **Estimate (B)** | **SE** | **t** | **p** |
| --- | --- | --- | --- | --- |
| **Model 1: Mid LC (Right)** |  |  |  |  |
| Intercept | 0.003 | 0.000 | 16.88 | < .001 |
| Linear: Mid LC (Right) | -0.002 | 0.002 | -0.89 | .378 |
| Quadratic: Mid LC (Right) | 0.002 | 0.003 | 0.73 | .466 |
| Premorbid intelligence: High | -0.001 | 0.000 | -2.92 | .004 |
| Linear: Mid LC × Premorbid intelligence: High | 0.001 | 0.002 | 0.31 | .756 |
| Quadratic: Mid LC × Premorbid intelligence: High | -0.001 | 0.003 | -0.44 | .658 |
|  |  |  |  |  |
| **Model 2: Rostral LC (Right)** |  |  |  |  |
| Intercept | 0.003 | 0.000 | 16.63 | < .001 |
| Linear: Ros LC (Right) | -0.000 | 0.002 | -0.03 | .979 |
| Quadratic: Ros LC (Right) | 0.005 | 0.004 | 1.46 | .146 |
| Premorbid intelligence: High | -0.001 | 0.000 | -3.03 | .003 |
| Linear: Ros LC × Premorbid intelligence: High | -0.000 | 0.002 | -0.00 | .998 |
| Quadratic: Ros LC × Premorbid intelligence: High | -0.005 | 0.004 | -1.40 | .164 |
|  |  |  |  |  |
| **Model 3: Caudal LC (Left)** |  |  |  |  |
| Intercept | 0.002 | 0.000 | 18.54 | < .001 |
| Linear: Cau LC (Left) | 0.002 | 0.001 | 1.13 | .263 |
| Quadratic: Cau LC (Left) | 0.003 | 0.001 | 2.29 | .024 |
| Premorbid intelligence: High | -0.000 | 0.000 | -2.58 | .011 |
| Linear: Cau LC × Premorbid intelligence: High | -0.002 | 0.002 | -0.78 | .437 |
| Quadratic: Cau LC × Premorbid intelligence: High | 0.001 | 0.002 | 0.25 | .805 |
|  |  |  |  |  |
| **Model 4: Mid LC (Left)** |  |  |  |  |
| Intercept | 0.003 | 0.000 | 18.04 | < .001 |
| Linear: Mid LC (Left) | -0.001 | 0.001 | -0.72 | .474 |
| Quadratic: Mid LC (Left) | 0.000 | 0.002 | 0.13 | .901 |
| Premorbid intelligence: High | -0.001 | 0.000 | -2.74 | .007 |
| Linear: Mid LC × Premorbid intelligence: High | -0.000 | 0.002 | -0.23 | .822 |
| Quadratic: Mid LC × Premorbid intelligence: High | 0.002 | 0.002 | 0.86 | .395 |
|  |  |  |  |  |
| **Model 5: Rostral LC (Left)** |  |  |  |  |
| Intercept | 0.003 | 0.000 | 17.86 | < .001 |
| Linear: Ros LC (Left) | 0.001 | 0.001 | 0.99 | .326 |
| Quadratic: Ros LC (Left) | -0.001 | 0.001 | -0.44 | .659 |
| Premorbid intelligence: High | -0.001 | 0.000 | -2.67 | .009 |
| Linear: Ros LC × Premorbid intelligence: High | -0.003 | 0.002 | -1.27 | .208 |
| Quadratic: Ros LC × Premorbid intelligence: High | -0.000 | 0.002 | -0.10 | .917 |

Note: Cau – caudal, Mid – middle, Ros – rostral, LC – locus coeruleus. Estimates reflect unstandardized coefficients. SE = standard error. Premorbid intelligence: Low is the reference group

##

# **Supplementary References**

1. Kievit, R. A. *et al.* Developmental cognitive neuroscience using latent change score models: A tutorial and applications. *Dev. Cogn. Neurosci.* **33**, 99–117 (2018).

2. Cattell, R. B. Abilities: Their structure, growth, and action. (1971).

3. Delorme, A. & Makeig, S. EEGLAB: an open source toolbox for analysis of single-trial EEG dynamics including independent component analysis. *J. Neurosci. Methods* **134**, 9–21 (2004).

4. Coito, A., Michel, C. M., Vulliemoz, S. & Plomp, G. Directed functional connections underlying spontaneous brain activity. *Hum. Brain Mapp.* **40**, 879–888 (2019).

5. Zaldivar, D., Goense, J., Lowe, S. C., Logothetis, N. K. & Panzeri, S. Dopamine is signaled by mid-frequency oscillations and boosts output layers visual information in visual cortex. *Curr. Biol.* **28**, 224–235 (2018).

6. Chaumon, M., Bishop, D. V. & Busch, N. A. A practical guide to the selection of independent components of the electroencephalogram for artifact correction. *J. Neurosci. Methods* **250**, 47–63 (2015).
